# The effect of 30° and 70° arthroscope use on femoral and tibial tunnel placement in arthroscopic anterior cruciate ligament reconstruction surgery

**DOI:** 10.1590/1806-9282.20251578

**Published:** 2026-06-19

**Authors:** Öner Kilinc, Fatih Günaydin, İdris Demirtas, Bülent Sakarya

**Affiliations:** 1 Mersin City Education and Research Hospital, Department of Orthopaedics and Traumatology – Mersin, Turkey.

**Keywords:** Anterior cruciate ligament reconstruction, Arthroscopy, Anterior cruciate ligament injuries, Arthroscopic surgery

## Abstract

**OBJECTIVE:**

The success of anterior cruciate ligament reconstruction depends on anatomical tunnel placement. The aim of this was to compare the effects of 30° and 70° arthroscopes on femoral and tibial tunnel positioning and their relationship with clinical outcomes.

**METHODS:**

This retrospective study analyzed 55 patients who underwent anterior cruciate ligament reconstruction between 2020 and 2024 (30° arthroscope, n=28; 70° arthroscope, n=27). All procedures were performed by the same surgeon using hamstring tendon grafts with suspensory femoral fixation and bioabsorbable tibial screws. Femoral and tibial tunnel positions were evaluated on postoperative computed tomography using the Bernard-Hertel and Staubli-Rauschning methods, respectively, with measurements performed by four orthopedic surgeons; clinical outcomes were assessed at ≥6 months using the International Knee Documentation Committee score.

**RESULTS:**

Femoral tunnels were significantly more posterior and distal in the 70° group (depth 29.4 vs. 38.7%; height 33.7 vs. 43.1%; both p<0.001). Tibial tunnel placement showed no significant difference (p=0.49). International Knee Documentation Committee scores were higher in the 70° group (89.2±5.4 vs. 84.6±6.8; p=0.02). Correlation and regression analyses confirmed femoral tunnel position as an independent predictor of functional outcome. Receiver operating characteristic curve analysis showed area under the curve values of 0.75 (95%CI 0.69–0.79) for depth and 0.71 (95%CI 0.67–0.78) for height.

**CONCLUSION:**

Use of a 70° arthroscope in anterior cruciate ligament reconstruction enables more anatomical femoral tunnel placement and improves functional outcomes, while tibial tunnel positioning is unaffected. Arthroscopic viewing angle should be regarded not only as a technical factor but also as a determinant of clinical success.

## INTRODUCTION

Anterior cruciate ligament (ACL) injuries are among the most common orthopedic problems that compromise the anterior and rotational stability of the knee. In active individuals, these injuries can lead to significant functional loss, and arthroscopic anatomical reconstruction surgery is considered the gold standard treatment^1^.

The anatomical placement of the graft is critical for surgical success^2,3^. During surgery, femoral and tibial tunnels are created using anatomical landmarks, guiding instruments, or fluoroscopy. The positioning of these tunnels plays a decisive role in both the biomechanical effectiveness of the reconstruction and the clinical outcomes^3,4^.

For assessing the anatomical position of the femoral tunnel, the “quadrant” method described by Bernard and Hertel is widely used^5^. The accuracy of tunnel placement largely depends on the arthroscopic viewing angle. While traditionally used 30° arthroscopes may be insufficient for visualizing certain anatomical areas, 70° arthroscopes provide clearer visualization of the posterior border of the femoral footprint, enabling surgeons to achieve a more anatomical tunnel placement^6,7^.

Tibial tunnel placement, on the other hand, is generally guided by fixed anatomical landmarks and is therefore less affected by the arthroscopic viewing angle^8^. Nonetheless, the anteroposterior positioning of the tibial tunnel is also crucial for the functional performance of the graft^9^.

This study aims to radiologically compare the effects of 30° and 70° arthroscopes on femoral and tibial tunnel placement in arthroscopic ACL reconstruction surgery and to evaluate their relationship with clinical scores.

## METHODS

In this retrospective comparative study, 55 patients who underwent arthroscopic ACL reconstruction between 2020 and 2024 were evaluated. All operations were performed by the same surgeon using ipsilateral hamstring tendon grafts and the manual anteromedial portal technique. The same anteromedial portal was used for both the 30° and 70° arthroscopes without any modification in portal placement; the 70° scope was employed solely to enhance visualization. The use of the 30° versus 70° arthroscope was not randomized; the preference for the 70° arthroscope reflected the surgeon’s technical choice to obtain a wider and clearer visualization of the femoral footprint, rather than any patient-related characteristics. Suspensory fixation devices were used for femoral tunnel fixation, and bioabsorbable screws were used for tibial tunnel fixation. Patients were divided into two groups based on the type of arthroscope used during surgery: Group A (30° arthroscope, n=28) and Group B (70° arthroscope, n=27).

Patients with a primary ACL tear, who had undergone postoperative knee computed tomography (CT) scans and had a minimum of 6 months of follow-up, along with complete clinical and demographic data, were included in the study. Exclusion criteria were revision surgery, concomitant ligament injuries, and inadequate CT imaging.

**Figure 1:**
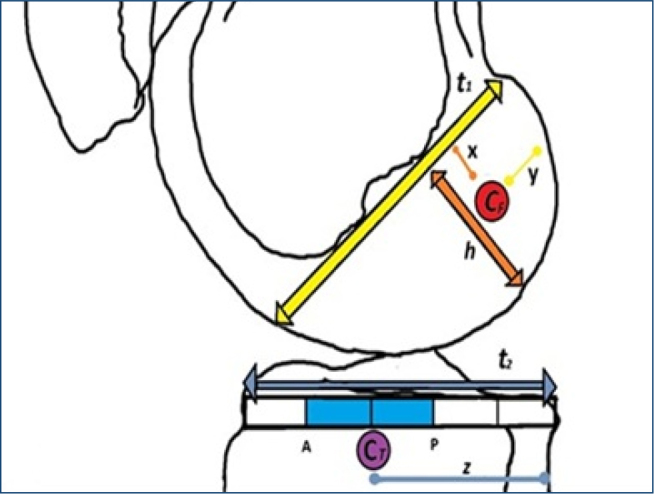
Diagram demonstrating the method used to measure the femoral and tibial tunnels. Evaluation of the femoral tunnel using the Bernard-Hertel method and the tibial tunnel using the Staubli-Rauschning method. Cf: Femoral tunnel center; x: distance to the Blumensaat line; y: distance to the posterior border; t1: length of the Blumensaat line; h: distance between the Blumensaat line and the distal border; Ct: Tibial tunnel center; A: anterior border of the ACL; P: posterior border of the ACL; t2: sagittal length of the tibia; z: distance from the tibial tunnel center to the posterior border; x/h: femoral height; y/t1: femoral depth; z/t2: tibial depth.

Femoral tunnel position was evaluated using the Bernard-Hertel quadrant method, while tibial tunnel position was analyzed using the Staubli-Rauschning method. The measurement procedures and the tunnel configurations of the groups are shown in Figure 1. Radiographic measurements were independently performed by four orthopedic surgeons. Any discrepancies among observers were resolved through a consensus-based approach, and the final value used in the analysis represented the shared agreement of all observers. At least 6 months postoperatively, all patients were evaluated with the International Knee Documentation Committee (IKDC) form, and the scores were analyzed.

The study was approved by the Mersin University Clinical Research Ethics Committee (Approval No: 2023/730, dated 01/11/2023), and informed consent was obtained from all patients. Based on power analysis performed with G*Power 3.1 software (α=0.05, 1–β=0.80, effect size d=0.8), a minimum of 26 patients per group was calculated to be required.

### Statistical analysis

Statistical analyses were conducted using SPSS v25.0 software. Data distribution was assessed with the Kolmogorov-Smirnov test. An independent-samples t-test was used for parametric variables, a chi-square test for categorical variables, and a p<0.05 was considered statistically significant.

## RESULTS

The 55 patients included in the study were divided into two groups according to the type of arthroscope used: Group A (30° arthroscope, n=28) and Group B (70° arthroscope, n=27). The groups were comparable in terms of age, sex, affected side, dominant side, and follow-up duration. The mean age was 27.4±5.8 years in Group A and 28.1±6.3 years in Group B, with no statistically significant difference (p=0.62). The proportion of male patients was 75% in Group A and 71% in Group B (p=0.77). The minimum follow-up period was 6 months for all patients, with mean follow-up durations of 9.1±2.3 months in Group A and 10.2±3.1 months in Group B. The dominant side was the right in 23 patients (82.1%) in Group A and 21 patients (77.8%) in Group B (p=0.69); the number of patients with the right knee affected was 17 (61%) and 18 (66%), respectively (p=0.73). The demographic, surgical, and clinical characteristics of the patients are summarized in Table 1.

**Table 1:** Comparison of demographic, surgical, and clinical characteristics between the 30° and 70° arthroscope groups.

Parameter	Group A (30°)	Group B (70°)	p-value
Age, years (mean±SD)	27.4±5.8	28.1±6.3	0.62
Male gender (n, %)	21 (75)	19 (71)	0.77
Right knee involvement (n, %)	17 (61)	18 (66)	0.73
Left knee involvement (n, %)	11 (39)	9 (34)	0.73
Dominant side (n, %)	23 (82.1)	21 (77.78)	0.69
Follow-up duration, months (mean±SD)	9.1±2.3	10.2±3.1	0.77
Femoral tunnel depth (mean±SD)	38.7±5.1	29.4±3.9	<0.001^*^
Femoral tunnel height (mean±SD)	43.1±4.8	33.7±4.2	<0.001^*^
Tibial tunnel position (mean±SD)	42.9±4.3	43.3±3.8	0.49
IKDC score (mean±SD)	84.6±6.8	89.2±5.4	0.02^*^

IKDC: International Knee Documentation Committee; SD: standard deviation.

*p<0.05.

**Figure 2:**
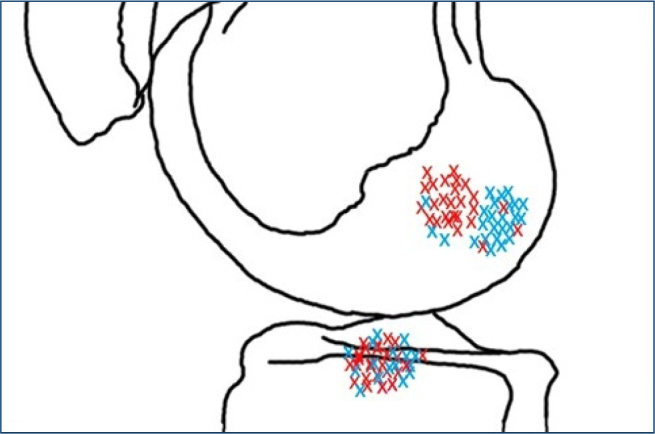
Illustration showing the tunnel configurations for the femur and tibia in the groups. Red crosses represent Group A (30° arthroscope), whereas blue crosses represent Group B (70° arthroscope).

When femoral tunnel positions were evaluated using the Bernard-Hertel quadrant method, the mean depth value of the femoral tunnel center was 29.4%±3.9 and the mean height value was 33.7%±4.2 in Group B. In Group A, these values were 38.7%±5.1 and 43.1%±4.8, respectively. Statistically significant differences were observed between the two groups in both depth and height parameters (p<0.001 for both). A shallower (i.e., more anatomical) femoral tunnel placement was positively correlated with higher IKDC scores. The locations of tunnel placements for the groups are demonstrated in Figure 2.

Tibial tunnel placement, evaluated using the Staubli-Rauschning method, revealed an anteroposterior position ratio of 42.9%±4.3 in Group A and 43.3%±3.8 in Group B, with no significant difference between groups (p=0.49). Postoperative IKDC scores were 84.6±6.8 in Group A and 89.2±5.4 in Group B, and this difference was statistically significant (p=0.02).

Femoral tunnel depth demonstrated an AUC of 0.75 (95%CI 0.69–0.79), and femoral tunnel height demonstrated an AUC of 0.71 (95%CI 0.67–0.78); both values were observed as predictors of clinical outcomes.

## DISCUSSION

This study aimed to evaluate the effects of 30° and 70° arthroscopes on femoral and tibial tunnel placement in arthroscopic ACL reconstruction surgery and to investigate the relationship of these placements with clinical outcomes. The findings demonstrated that the use of a 70° arthroscope provides significant advantages, particularly with respect to femoral tunnel positioning. In the 70° arthroscope group, the femoral tunnel was located significantly more posterior and distal, which corresponds to a more anatomical positioning. This difference can be attributed to the ability of the wide-angle arthroscope to provide clearer visualization of the posterior border of the femoral footprint. In the literature, Bedi et al. reported that the 70° arthroscope is superior to the conventional 30° arthroscope in visualizing anatomical structures behind the intercondylar notch^6^. Similarly, pioneering studies by Gillquist and Schneider emphasized the direct impact of portal placement on the quality of arthroscopic visualization^7,8^.

From a clinical perspective, this anatomical advantage was reflected in patient outcomes. IKDC scores were significantly higher in patients operated on with a 70° arthroscope, indicating that more anatomical femoral tunnel placement supports better postoperative functional recovery. Correlation analyses further confirmed this relationship. Significant positive correlations were found between femoral tunnel depth and height parameters and IKDC scores. Regression analyses revealed that femoral tunnel placement was an independent predictor. These findings are consistent with Zhang et al., who emphasized the association between femoral tunnel position, knee stability, and functional outcomes^3^.

Our study demonstrated that femoral tunnel parameters showed an association with IKDC outcomes. Receiver operating characteristic (ROC) analysis indicated that femoral tunnel depth (area under the curve [AUC]=0.75, 95%CI 0.69–0.79) and height (AUC=0.71, 95%CI 0.67–0.78) had only moderate discriminative capacity. These findings suggest that a more anatomical femoral tunnel placement may contribute to early functional improvement, although the predictive value of these parameters remains limited and should be interpreted with caution.

On the other hand, no significant difference was observed between groups in terms of tibial tunnel placement. This can be explained by the fact that tibial tunnels are guided by more fixed and predictable anatomical landmarks and that the arthroscopic viewing angle has limited influence on the posterior tibial area. Measurements obtained using the percentage method described by Staubli and Rauschning yielded similar results in both groups^10^. Nevertheless, opposing views in the literature suggest that tibial tunnel placement is not constant. Iriuchishima and Goto demonstrated that the position of the tibial spine is a determinant of tunnel orientation, while Pedneault and colleagues reported that, in patients undergoing anatomic reconstruction, deviations may occur due to these anatomical variations^11,12^. In addition, Montreuil et al. suggested the use of biplanar stereoradiography and grid systems for more precise evaluation of tibial tunnel placement^13^.

It should also be noted that tibial tunnel drilling can affect not only graft positioning but also meniscal integrity. In a cadaveric study, LaPrade et al. demonstrated that placing the tunnel over the anterior root of the lateral meniscus may compromise its structural integrity^14^. Therefore, tibial tunnel placement should be carefully considered not only in terms of graft trajectory but also in relation to surrounding structures. In this context, the 70° arthroscope was also observed to be more effective in visualizing the anterior margins of the menisci.

In our study, ipsilateral hamstring tendon grafts were used in all patients, with suspensory fixation on the femoral side and bioabsorbable screws on the tibial side. The studies of Hatipoğlu et al. and Alomari et al. have shown that this graft type yields reliable and successful clinical outcomes^15,16^. Previous studies also reported that bioabsorbable screws provide similar clinical success rates to metallic screws and demonstrate high biocompatibility^17^. Samuelsson et al., in a systematic review, emphasized the importance of maintaining homogeneity in graft and fixation methods when comparing surgical techniques^18^. Accordingly, the use of a uniform graft and fixation method in our study allowed for an isolated evaluation of the effect of arthroscopic viewing angle on femoral tunnel placement, thereby enhancing the reliability of the group comparison and strengthening the relationship between findings and arthroscope type.

Although IKDC scores were statistically higher in the 70° arthroscope group, the magnitude of this early difference may have limited clinical impact. However, the more anatomical femoral tunnel positioning obtained with the 70° arthroscope may contribute to functional improvement, which could become more evident with longer-term follow-up.

This study has certain limitations. First, the retrospective design and limited sample size restrict the generalizability of the findings. Although the power analysis confirmed that the sample size was adequate to detect major differences in femoral tunnel parameters, the overall number of patients was still limited. Therefore, smaller or more subtle differences — particularly in secondary outcomes such as tibial tunnel positioning — may not have been fully captured. This may restrict the generalizability of the results. Due to the retrospective design, adjustments for variables such as preoperative activity level, concomitant meniscal pathology, or rehabilitation protocols were not possible. However, the demographic and surgical similarities between groups likely minimized their potential influence. All procedures were performed by a single surgeon with extensive arthroscopic experience, and the use of the 70° arthroscope reflected a technical preference rather than a learning-curve effect. The mean follow-up period of 9–10 months reflects early postoperative outcomes and does not allow assessment of long-term functional results or graft integrity. Longer-term follow-up studies are needed to better understand these effects.

The exclusive use of hamstring autografts and uniform fixation methods minimizes surgical variability but limits the generalizability of the findings to other graft choices such as bone– patellar, tendon–bone, or allografts. Graft healing and biological integration could not be evaluated with advanced imaging modalities such as MRI. As suggested by Bird and Steiner, three-dimensional CT and high-resolution imaging techniques could provide a more precise analysis of tunnel placement and graft integrity^19,20^. In addition, the use of a 70° arthroscope may present technical challenges for surgeons, including a learning curve, portal positioning, and operative time. Therefore, future prospective, randomized, multicenter studies that incorporate variables such as surgeon experience, operative time, graft type, and imaging modalities are warranted to enhance the reliability and clinical applicability of the findings.

## CONCLUSION

This study demonstrated that the use of a 70° arthroscope in arthroscopic ACL reconstruction allows for more anatomical femoral tunnel placement, which in turn translates into improved clinical outcomes. The improvement in femoral tunnel positioning was associated with higher IKDC scores. In contrast, tibial tunnel placement was not influenced by arthroscope type. These findings indicate that the arthroscopic viewing angle is an important factor affecting not only surgical technique but also patient outcomes. The enhanced visualization provided by the 70° arthroscope may contribute to more anatomical tunnel placement.

Future studies with larger samples, prospective designs, and comprehensive analyses—including surgeon experience and long-term results—will be beneficial in validating these findings.

## Data Availability

The datasets generated and/or analyzed during the current study are available from the corresponding author upon reasonable request.
